# Unraveling the mutations of the core histone proteins in neurodevelopmental disorders: Overview of Histonopathies

**DOI:** 10.1192/j.eurpsy.2026.10738

**Published:** 2026-07-31

**Authors:** N. Bouayed Abdelmoula, B. Abdelmoula

**Affiliations:** LR23ES07 Genomics of Signalopathies at the service of Precision Medicine, Medical University of Sfax, Sfax, Tunisia

## Abstract

**Introduction:**

Chromatinopathies encompass a group of neurodevelopmental disorders caused by genetic alterations affecting the structure and regulation of chromatin. Among them, histonopathies result from germline mutations in the genes encoding histone proteins, which form the fundamental structural unit of chromatin (the nucleosomic fiber), essential for DNA compaction and epigenetic modulation of gene expression.

**Objectives:**

This review aims to synthesize current knowledge on histonopathies and to outline clinical and psychiatric manifestations, for guiding early recognition and genetic diagnosis in affected individuals.

**Methods:**

We have carried out a review of recent publications on histonopathies, with an emphasis on the molecular mechanisms involved, the associated syndromes (in particular the HIST1H1E syndrome, Bryant-Li-Bhoj and Tessadori-Bicknell-van Haaften syndromes) as well as the clinical manifestations including facial dysmorphy and psychiatric features.

**Results:**

The mutations leading to histonopathies affect various histone genes (HIST1H1E, HIST1H2AL, HIST1H3D, HIST3H3, among others), disrupting chromatin dynamics and gene expression. Clinically, these mutations result in a spectrum of neurodevelopmental disorders with intellectual retardation, language impairment, motor delay, often marked but heterogenous craniofacial dysmorphism, as well as frequent psychiatric disorders such as autism spectrum disorders, anxiety, and attention disorders. Phenotypic and age-dependent evolutionary variability complicates clinical recognition, highlighting the need for molecular diagnosis by next-generation sequencing essential.

**Conclusions:**

Understanding the epigenetic mechanisms and associated syndromes is essential to raise clinician awareness and facilitate early referral for appropriate genetic exploration. In the absence of specific treatment, multidisciplinary management targeting both neurodevelopmental and psychiatric aspects can improve the quality of life of patients. These findings also pave the way for future therapeutic approaches based on epigenetic modulation.

**Disclosure of Interest:**

None Declared

